# Impending Respiratory Failure in Miller Fisher Syndrome: A Report of a Unique Case

**DOI:** 10.7759/cureus.71938

**Published:** 2024-10-20

**Authors:** Sondos Badran, Johnny S Randhawa, Renard Jerome, David Karp, Sarkis Arabian

**Affiliations:** 1 Internal Medicine, Arrowhead Regional Medical Center, Colton, USA; 2 Critical Care, Arrowhead Regional Medical Center, Colton, USA

**Keywords:** acute hypercapnic respiratory failure, albuminocytologic dissociation, anti-gq1 antibody, guillain-barré syndrome (gbs), intubation, miller fisher syndrome (mfs)

## Abstract

Miller Fisher syndrome (MFS) is a variant of Guillain-Barré syndrome (GBS), where the body's immune system erroneously attacks its own nerves. It typically presents with a triad of symptoms: ataxia, ophthalmoplegia, and areflexia. These symptoms often develop rapidly, usually within a few days after a viral or bacterial infection, most commonly following respiratory or gastrointestinal illnesses. The diagnosis of MFS involves clinical examination, electromyography (EMG), nerve conduction studies, and sometimes lumbar puncture to analyze cerebrospinal fluid (CSF). Treatment primarily focuses on supportive care and symptomatic management, with more severe cases requiring intravenous immunoglobulin (IVIG) or plasma exchange (PLEX). Most patients experience spontaneous recovery over several weeks to months, although some may require rehabilitative therapy to regain full function.

We present a case of a 47-year-old female with no known past medical history who presented to the emergency department with complaints of left-sided facial droop, dysphagia, pressure-like chest pain, and progressively worsening bilateral upper and lower extremity weakness. Computed tomography (CT) of the head without intravenous contrast was unremarkable for any intracranial abnormalities. Lumbar puncture was performed and was grossly unremarkable for albuminocytologic dissociation; however, CSF serology was significant for positive anti-GQ1 antibody. On the first day of hospitalization, the patient was noted to have increased work of breathing and was subsequently intubated for acute hypercapnic respiratory failure. The patient received therapy with IVIG and PLEX and eventually had a tracheostomy and a percutaneous endoscopic gastrostomy (PEG) tube was placed. The patient was then discharged to a long-term acute care facility (LTAC) with outpatient neurology follow-up. This case aims to emphasize the importance of physical exams and clinical intuition when guiding diagnostics and interventions for complex medical conditions with atypical presentation, such as our case of MFS.

## Introduction

Miller Fisher syndrome (MFS) is a rare variant of Guillain-Barré syndrome (GBS) with an incidence of 1-2 cases per 100,000 worldwide [1]. In Western countries, MFS accounts for 1-5% with a male predominance. Around 80% of patients present with a classic triad of symptoms such as ataxia, areflexia, and ophthalmoparesis, while others may present with atypical symptoms. Around 30% of patients can present with atypical symptoms including headache, delayed facial palsy, divergence insufficiency, and taste impairment [2]. Delayed facial palsy occurs in 8% of patients. Patients usually have a full recovery of symptoms within six months.

Although the pathogenesis of MFS is not well understood, molecular mimicry is thought to play a role. The immune system activation induces the production of autoantibodies, especially against anti-ganglioside and anti-GQ1b antibodies. Anti-GQ1b antibody is found in over 80% of patients with MFS [3]. It is 85% sensitive and 100% specific for MFS [1]. 

Here, we present the case of a 47-year-old female patient with no past medical history, who presented with bilateral upper and lower extremity weakness as the primary manifestations of MFS. The patient underwent treatment with intravenous immunoglobulin (IVIG) or plasma exchange (PLEX), which unfortunately did not improve the patient's recovery. 

## Case presentation

A 47-year-old female with no past medical history presented to the emergency department with left-sided facial droop, progressively worsening bilateral upper and lower extremity weakness, dysphagia, and pressure-like chest pain. The patient stated she was sick one week prior with subjective fevers and chills. On review of symptoms, she denied any recent symptoms such as diarrhea, abdominal pain, rhinorrhea, or cough with productive mucus. She took ibuprofen with symptomatic improvement. After a couple of days, she also started experiencing numbness and tingling sensations in her hands and feet while showering. The patient could not stand or walk. The following day, the patient started experiencing facial droop.

In the emergency department, vitals showed a temperature of 36℃ (96.8℉), blood pressure 148/85 mmHg, heart rate 99 beats per minute, respiratory rate 22, and an oxygen saturation of 94% on room air. On physical examination, the patient had left-sided lower facial droop, left-sided ptosis, dysarthria, diffusely decreased deep tendon reflexes, and absent Babinski sign bilaterally. Muscle strength in the left upper extremity was 1/5, right upper extremity 2/5, and bilateral lower extremity 0/5. Computed tomography (CT) of the head was done to rule out any acute hemorrhage, which was unremarkable. Labs were unremarkable except for an elevated creatinine kinase of 217 units/liter (Table 1). Neurology was consulted and recommended obtaining the following: a lumbar puncture, negative inspiratory force, tidal volume monitoring, and magnetic resonance imaging (MRI) of the cervical and lumbar spine. The patient was then admitted for acute quadriparesis. 

**Table 1:** Laboratory values on admission BUN: Blood urea nitrogen

Laboratory Study	Reference Values	Measured Values
White blood cells	4.5-11.2 x 10^3 ^/μL	9.7
Red blood cells	4-5.2 x 10^6^/μL	4.48
Hemoglobin	11.5-15.5 g/dL	13.1
Haematocrit	36-46%	39
Mean corpuscular volume	80-100 fL	87
Platelets	120-360 x 10^3 ^/μL	355
Sodium	135-148 mmol/L	140
Potassium	3.5-5.5 mmol/L	3.9
Chloride	98-110 mmol/L	103
CO_2_	24-34 mmol/L	24
BUN	8-20 mg/dL	10
Creatinine	0.5-1.5 mg/dL	0.66
Glucose	65-125 mg/dL	110
Calcium	8.5-10.5 mg/dL	9.3
Total creatinine kinase	30-170 U/L	217

On the first day of hospitalization, the patient started having increased work of breathing with a respiratory rate of 25-30 breaths per minute. Vital capacity decreased from 350 milliliters to 150 milliliters. Negative inspiratory force (NIF) was -6 centimeters of water with a 2-liter nasal cannula (Table 2). The patient was emergently intubated due to acute hypercapnic respiratory failure and upgraded to the intensive care unit. An initial venous blood gas showed pH 7.39, partial carbon dioxide 47, and partial oxygen <49. After intubation, the patient had an arterial blood gas drawn, which showed pH 7.37, partial carbon dioxide 41, and partial oxygen 389.

**Table 2:** Negative inspiratory force throughout hospitalization

Day	Time	Negative Inspiratory Force (cm of H_2_O)	Vital Capacity (mL)
10/2/2023	00:14 AM	-10	350
10/2/2023	12:40 PM	-8	250
10/2/2023	13:35 PM	-6	150

Lumbar puncture was later performed to rule out meningitis, multiple sclerosis, and other viral etiologies, such as syphilis and West Nile virus. Spinal fluid shows elevated glucose at 80, protein 41, red blood cells at 3,000, and white blood cells at 0 (Table 3). Cerebrospinal fluid (CSF) was pink and hazy in characteristic. CSF fluid analysis was negative for any bacteria or viruses.

**Table 3:** Cerebrospinal fluid analysis obtained from lumbar puncture

	Lab Value
Protein (mg/dL)	41
Glucose (mg/dL)	80
Red blood cells (cells/mm^3^)	3000
White blood cells (cells/uL)	0.0
Opening pressure (cm of H_2_O)	11

**Figure 1:**
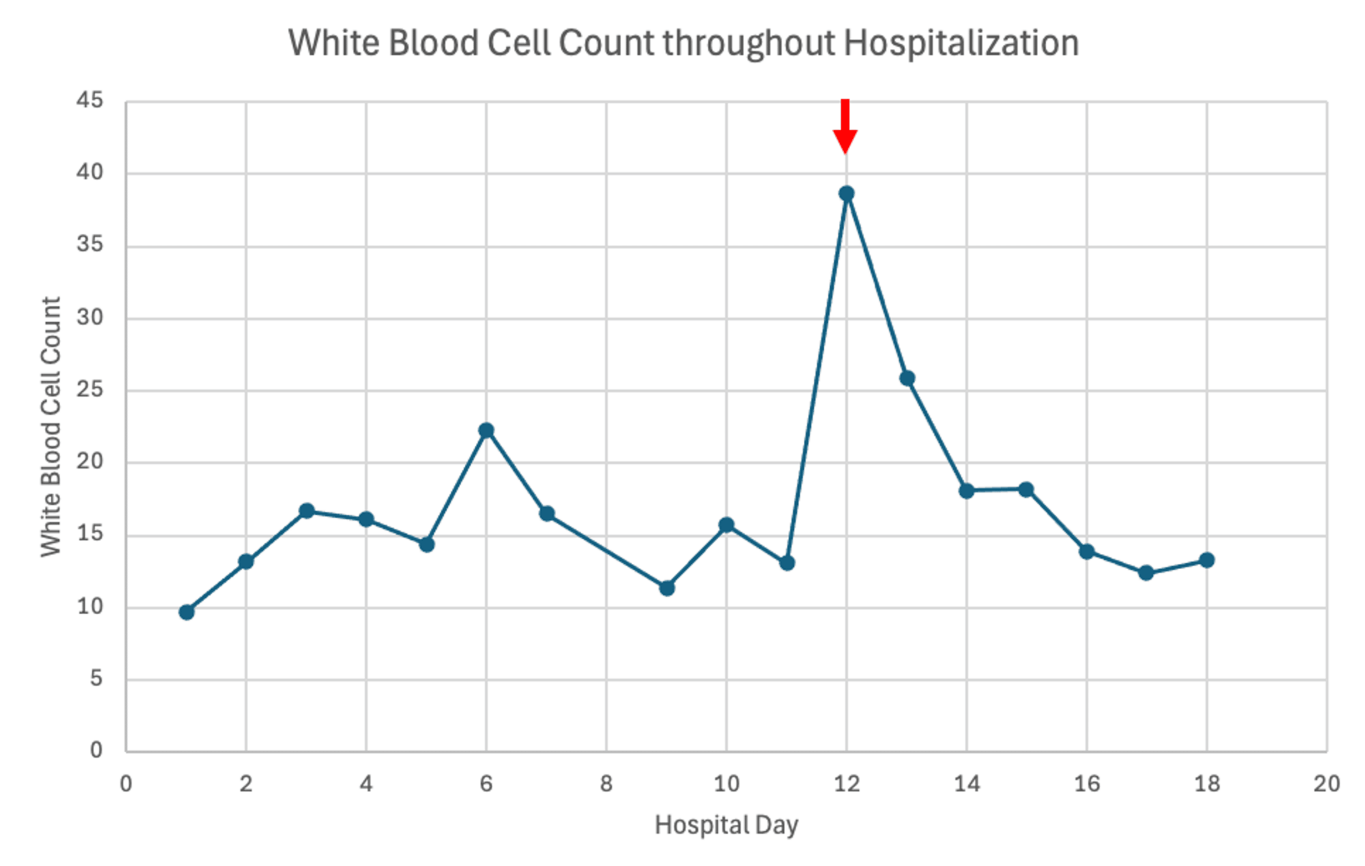
White blood cell count throughout hospitalization. The red arrow indicates when the second session of PLEX occurred. PLEX: Plasma exchange

Due to high clinical suspicion of GBS versus myasthenia gravis, the patient was started on IVIG. The patient received three days of 0.4 grams/kilogram of IVIG without any improvement. One day after the initiation of IVIG, PLEX was started. The patient had five total sessions of PLEX. During the second session of PLEX, the patient’s white blood cell count (WBC) increased to 38.7 (Figure 1). WBC was monitored. An infectious disease specialist was consulted, who concluded that the elevated WBC was likely reactive from PLEX. The patient’s WBC eventually downtrended. Due to the downtrending WBC, infectious etiology was less likely and hence not pursued. The WBC elevation was attributed to a reactive etiology.

During this time, serum antibody testing was sent out, including anti-acetylcholine receptor antibody, aldolase, anti-neutrophil cytoplasmic antibody (ANCA), antinuclear antibody (ANA), anti-muscle specific tyrosine kinase (MuSK) antibody, and anti-GQ1 antibody. MRI of the brain, cervical spine, thoracic spine, and lumbar spine was done to rule out any other etiologies such as multiple sclerosis of the spine. All MRIs were unremarkable for evidence of multiple sclerosis. Antibody testing was significant for ANA 1:80 titer and a positive anti-GQ1 antibody. All other antibody tests were negative.

Due to the unknown cause of MFS, the infectious disease specialist recommended obtaining additional serum IgG and IgM West Nile, cytomegalovirus polymerase chain reaction (PCR), human immunodeficiency virus antibody, Epstein-Barr virus IgG and IgM serology, acute tick panel test, arbovirus panel test, *Rickettsia typhi* antibody, and gastroenterology pathogen panel. The acute tick panel test consists of *Ehrlichia chaffeensis*, *Borrelia miyamotoi*, *Babesia microti*, and *Anaplasma phagocytophilum* protein reaction chain testing. The arbovirus panel consists of IgG and IgM of California encephalitis, Eastern equine encephalitis virus, Western equine encephalitis virus, and St. Louis encephalitis virus. All the infectious work-up was negative.

During her hospitalization, the patient worked closely with physical therapy, speech and language pathology, and occupation therapy without any significant improvement in her ability to move her extremities. As a result, a nerve conduction study was done, which showed severely abnormal motor and sensory function. The patient had a gastric tube placed for long-term feeding and nutritional support. Due to the patient being on ventilation for almost two weeks with no improvements in spontaneous breathing trials, a tracheostomy was also placed. The patient was then discharged to a long-term acute care (LTAC) facility. 

At the LTAC, the patient continued to be mechanically ventilated. The patient had a follow-up with a neurologist two months after PLEX treatment. The patient had mild improvement in mobility. The patient was able to move her first digits of bilateral hands. The patient also had 4 out of 5 muscle strength in distal bilateral lower extremities. However, the deep tendon reflex remained 0 in the upper and lower extremities. The patient was eventually weaned down from her ventilation settings and placed on a Passy Muir Valve (PMV) eight months after her initial diagnosis of MFS. Furthermore, the patient was able to regain mobility in her lower extremities at eight months. The patient is now able to walk with a front-wheel walker. The patient was discharged from LTAC nine months after hospital discharge. 

## Discussion

MFS is one entity from the group of GQ1b syndromes, which include ophthalmoplegia, ataxia, areflexia [4]. Patients may present with hyporeflexia, loss of vibratory and light touch sensation, bilateral dilated pupils, and pharyngeal involvement [5]. Around 30% of patients can present with atypical symptoms including headache, delayed facial palsy, divergence insufficiency, and taste impairment [2]. Delayed facial palsy occurs in 8% of patients. Clinical features can help with the diagnosis. However, to confirm the diagnosis, lumbar puncture is commonly performed. Increased protein without an increase in WBC in the CSF is a classical laboratory finding of MFS, called albuminocytologic dissociation [6]. Albuminocytologic dissociation is seen in 50-66% of patients with MFS following their first week of symptoms. The percentage increases to 75% during the third week since the onset of symptoms. Furthermore, ANA is an infrequent presentation of MFS and is found in a few case reports [7]. Respiratory weakness occurs in 10-30% of patients. However, 5-10% require mechanical ventilation due to respiratory weakness [8]. 

In our case report, a 47-year-old patient demonstrated some classical signs seen in MFS including ophthalmoplegia, ataxia, and areflexia. Our patient had atypical signs including delayed facial palsy. The patient had a positive ANA, which is infrequently found in MFS. Anti-GQ1b was positive in the patient, which confirmed the diagnosis of MFS. The patient also demonstrated uncommon signs including her female gender, which is uncommon for MFS. Additionally, the lumbar puncture did not show any albuminogenic dissociation. Repeat lumbar puncture was suggested to get more CSF, however, the patient’s family declined. Furthermore, the patient was in acute respiratory failure, requiring intubation and then a tracheostomy tube. The patient was dependent on ventilation for approximately eight months before being transitioned to a PMV. 

Cochrane reviews have shown that IVIG and plasma exchange decrease stay in the intensive care unit [9]. Despite IVIG and plasma exchange, patients may have persistent fatigue and approximately 9-13% may still need aid to walk after MFS onset. Case reports have shown that IVIG and/or PLEX should correlate to a resolution of symptoms within 35 days for ophthalmoplegia, three months for areflexia, and complete resolution of triad within six months [1]. 

A limitation of this case is the use of PLEX after IVIG. There has been some controversy about this topic as a Lancet article in 2008 stated that PLEX after IVIG would cause the PLEX to wash out the IVIG previously administered. However, since that paper, many articles have looked at the administration of PLEX and IVIG, specifically in GBS [10]. Oczko-Walker et al. demonstrated no improvement in patients having PLEX after IVIG. However, there was an increase in cost and hospitalization duration [11]. Buzzigoli et al. discussed PLEX after IVIG in GBS in a 78-year-old patient [12]. This combination improved respiratory function and peripheral muscle strength, suggesting the benefit of PLEX after IVIG in refractory cases. In our case, the patient did not initially improve with IVIG, and PLEX was decided to be started. 

Despite treatment with PLEX and IVIG, our patient still required mechanical ventilation. Our patient had a longer extensive course in the resolution of symptoms. She first started gaining mobility in her lower extremities at nine months after presenting to the hospital. Hence, this paper highlights the importance of recognition of MFS to facilitate prompt treatment in order to enhance patient recovery and activities of daily living.

## Conclusions

In conclusion, the characteristic triad of ataxia, areflexia, and ophthalmoparesis is the hallmark of MFS, a variant of GBS. While the majority of patients recover completely with early recognition of MFS and treatment with IVIG or PLEX, some may endure protracted and severe symptoms that necessitate prolonged mechanical ventilation and rehabilitation. This case emphasizes an atypical presentation of MFS, highlighting the importance of relying on physical exams and clinical judgment to guide medical management. To our knowledge, this is the first case of MFS in which the patient did not improve after IVIG and plasmapheresis treatments and continues to need assistance in her mobility. This case underscores the importance of early diagnosis and treatment to enhance recovery outcomes for MFS patients.

## References

[1] Noioso CM, Bevilacqua L, Acerra GM (2023). Miller Fisher syndrome: an updated narrative review. Front Neurol.

[2] Jung JH, Oh EH, Shin JH, Kim DS, Choi SY, Choi KD, Choi JH (2019). Atypical clinical manifestations of Miller Fisher syndrome. Neurol Sci.

[3] Dagklis IE, Papagiannopoulos S, Theodoridou V, Kazis D, Argyropoulou O, Bostantjopoulou S (2016). Miller-Fisher syndrome: are Anti-GAD antibodies implicated in its pathophysiology?. Case Rep Neurol Med.

[4] Wu X, Wang Y, Xi ZQ (2023). Clinical and antibodies analysis of anti-GQ1b antibody syndrome: a case series of 15 patients. Acta Neurol Belg.

[5] Truong J, Conley J, Ashurst J (2020). Miller-Fisher syndrome: a case report and review of the literature. Clin Pract Cases Emerg Med.

[6] Gupta SK, Jha KK, Chalati MD, Alashi LT (2016). Miller Fisher syndrome. BMJ Case Rep.

[7] Mercado C, Perez-Rueda M (2022). An atypical case of Miller Fisher syndrome with multiple autoimmunity. Neuroophthalmology.

[8] Alqahtani SA, Alnaami I, Shubaili M, Alqahtani MS (2020). Unusual presentation of Miller Fisher variant syndrome. Bahrain Med Bull.

[9] Doets AY, Hughes RA, Brassington R, Hadden RD, Pritchard J (2020). Pharmacological treatment other than corticosteroids, intravenous immunoglobulin and plasma exchange for Guillain-Barré syndrome. Cochrane Database Syst Rev.

[10] van Doorn PA, Ruts L, Jacobs BC (2008). Clinical features, pathogenesis, and treatment of Guillain-Barré syndrome. Lancet Neurol.

[11] Oczko-Walker M, Manousakis G, Wang S, Malter JS, Waclawik AJ (2010). Plasma exchange after initial intravenous immunoglobulin treatment in Guillain-Barré syndrome: critical reassessment of effectiveness and cost-efficiency. J Clin Neuromuscul Dis.

[12] Buzzigoli SB, Genovesi M, Lambelet P, Logi C, Raffaelli S, Cattano D (2010). Plasmapheresis treatment in Guillain-Barré syndrome: potential benefit over intravenous immunoglobulin. Anaesth Intensive Care.

